# Progress in understanding Legg–Calvé–Perthes disease etiology from a molecular and cellular biology perspective

**DOI:** 10.3389/fphys.2025.1514302

**Published:** 2025-02-17

**Authors:** Xinda Zheng, Zhuqing Dong, Xiaofei Ding, Qian Huang, Shengping Tang, Yuchen Zhang, Boxiang Li, Shijie Liao

**Affiliations:** ^1^ Department of Trauma Orthopedic and Hand Surgery, The First Afliated Hospital of Guangxi Medical University, Nanning, Guangxi, China; ^2^ Guangxi Key Laboratory of Regenerative Medicine, Orthopaedic Department, The First Affiliated Hospital of Guangxi Medical University, Nanning, Guangxi, China; ^3^ Department of Orthopedics, Minzu Hospital of Guangxi Zhuang Autonomous Region, Nanning, Guangxi, China

**Keywords:** perthes disease, etiology, pathogenesis, molecular biology, cellular biology

## Abstract

Legg–Calvé–Perthes disease (LCPD) is a hip disease caused by ischemia of the femoral epiphysis in children, which occurs in children aged 4–8 years (mean 6.5 years), with a male-to-female ratio of about 4:1. The disease has been reported for more than 100 years, but its etiology has not been elucidated. In recent years, a considerable amount of research has been carried out on the etiology of the disease, and the development of the disease is believed to involve a variety of molecular biological alterations, such as the *COL2A1* mutation, which may be one of the causes of necrotic collapses of the epiphyseal cartilage matrix in LCPD. Tissue factor V Leiden mutation and insulin-like growth factor (IGF-1) abnormalities have also been reported in LCPD, but most theories need further confirmation. The in-depth study of LCPD cell biology has facilitated the suggestion regarding structural and/or functional abnormalities of microvascular endothelial cells in LCPD. This conjecture is supported by epidemiological and clinical evidence. Abnormal activation of osteoclasts, ischemic damage to epiphyseal cartilage, and activation of the bone marrow immune system all play important roles in the onset and progression of the disease. In this paper, we review the previous basic studies on LCPD and give an overview from the molecular biology and cell biology perspectives.

## 1 Introduction

**FIGURE 1:**
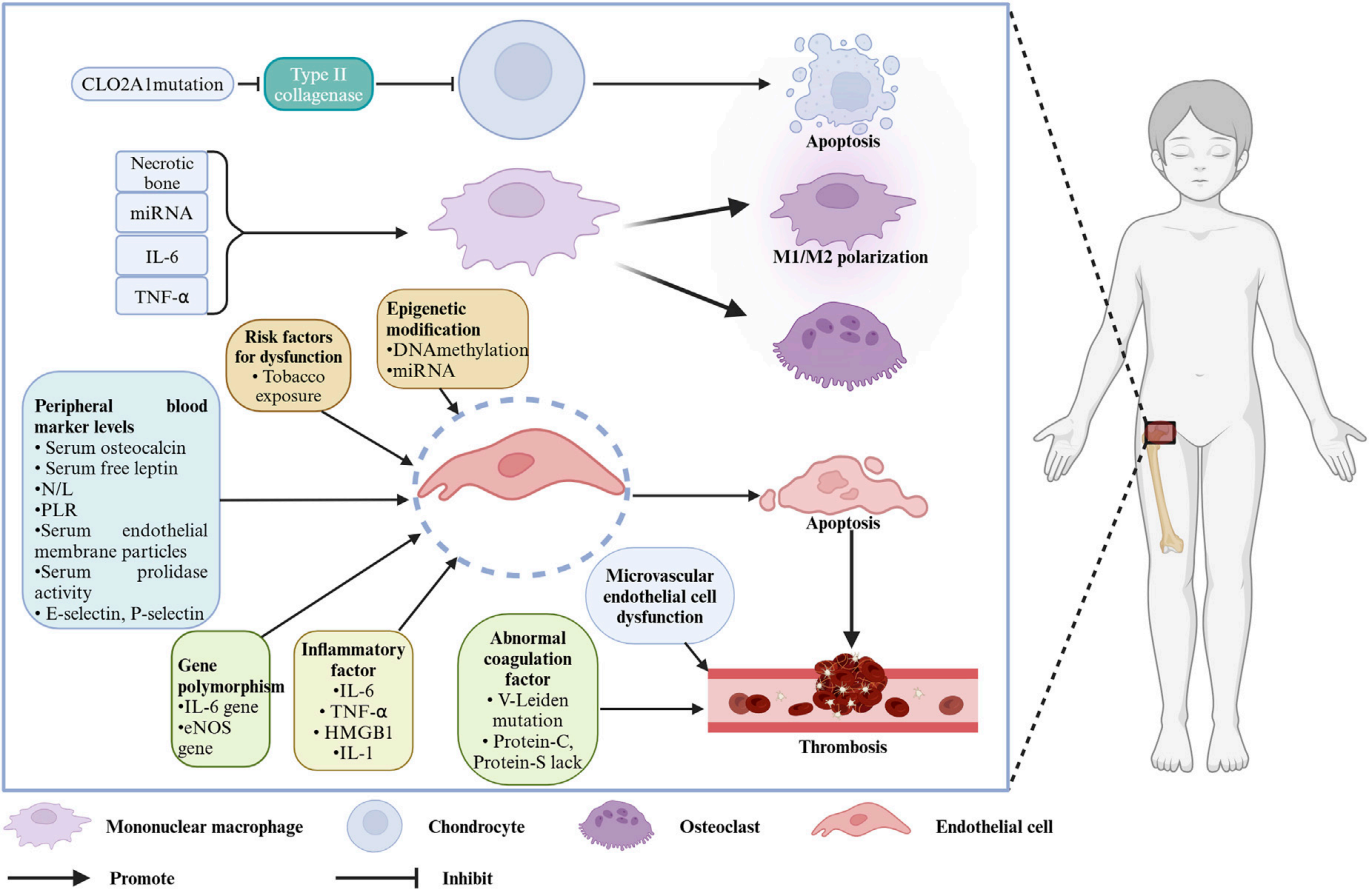
Molecules and cells associated with the pathogenesis of LCPD. Created with BioRender.com.

Legg–Calvé–Perthes disease (LCPD) was first described in 1910 by three researchers, Legg (Legg, 2006), Calvé (Calve, 2006), and Perthes (Perthes, 2012). LCPD is also referred to as ischemic necrosis of the femoral head epiphysis in children. The disease commonly occurs in children aged 4–8 years (average 6.5 years), with an incidence of 0.4/100,000 to 29/100,000, and a male-to-female ratio of about 4:1. Its incidence is associated with regional socioeconomic status, gender, and race (Loder and Skopelja, 2011; Kessler and Cannamela, 2018). The natural course of LCPD is approximately 34 months, and after healing of femoral head necrosis, varying degrees of deformity may persist, with patients at risk of early-onset arthritis after skeletal maturation (Catterall, 1971; Mose, 1980; Stulberg et al., 1981). Clinically, the etiology of LCPD is often not well understood, imposing a significant physical and psychological burden on children, making understanding the cause of this disease essential. Although this disease has been reported for over a century, extensive etiological research has been carried out by many scholars, but the exact cause remains uncertain. Traditional perspectives attribute the disease to factors such as low socioeconomic status, maternal or childhood tobacco exposure (Daniel et al., 2012; Perry et al., 2017), endocrine abnormalities like insulin-like growth factor 1 (IGF-1) dysfunction (Baş et al., 2015), and anatomical issues such as venous congestion in the femoral head, arterial occlusion, vascular anomalies, and congenital factors like *in-utero* effects (Perry et al., 2012a). Detailed discussions of these potential causes have been provided by scholars. For example, Gao et al. (2020) explained the relationship between passive smoking and LCPD from the perspective of different passive smoking types (e.g., paternal smoking, maternal smoking, tobacco exposure during pregnancy). The etiology of LCPD was fully elaborated by Rodríguez-Olivas et al. (2022), who suggested that LCPD may be correlated with factors such as the environment, race, and coagulation. Some scholars also found that some molecular markers related to the pathogenesis of LCPD, including VEGF, *eNOS*, and *IL-6*, may be involved in the disease progression of LCPD, potentially helping the treatment and diagnosis to some extent (Spasovski et al., 2023) (as shown in Figure 1).

In recent years, significant progress has been made in etiological research on LCPD. Building on these reviews, we aim to summarize the current research status from a molecular biology perspective, focusing on aspects like gene mutations, epigenetic modifications, and gene polymorphisms. Molecular-level changes may further affect cell function, so we summarize the progress in pathogenesis research from a cellular biology perspective, particularly focusing on microvascular endothelial cell structural and/or functional abnormalities in the hip joint, abnormal osteoclast activation, ischemic damage to epiphyseal cartilage, and the bone marrow immune system. This deepens the understanding of the disease and provides a reference for further basic research.

## 2 Molecular biology

The molecular level is the lowest level at which we currently understand the disease and may be the cause of the disease. Previous reports of changes in the molecular level of LCPD may include *COL2A1* mutation, tissue factor V Leiden mutation, and IGF-1dysfunction, but these molecular changes as independent factors to cause the disease remain controversial, including sample selectivity bias, and small sample size. In recent years, significant progress has been made in the molecular biology of this disease. We therefore attempt to summarize the advances in molecular biological research from the perspectives of gene mutations, epigenetic modifications, and gene polymorphisms, to elucidate the molecular-level pathological changes in LCPD. The main findings of the included articles are summarized (Table 1).

**TABLE 1:** Main findings of the included case-control studies.

Molecular biology	Ref.	Source of specimen	Subjects	Results/Conclusions
*COL2A1*	Li et al. (2014)	Leukocyte genomic DNA (human)	A four-generation family/45 members/LCPD or ANFH	This study identified a novel missense mutation (c. 1888 G>A) in the *COL2A1* gene, leading to type II collagenopathy, which manifests as LCPD or ANFH.
	Su et al. (2010)	Cartildral tissue of femoral head (human)	A five-generation family/42 members/novel type II collagen disease	The p.Gly1170Ser mutation in *COL2A1* leads to significant structural changes in joint cartilage, resulting in early-onset hip osteoarthritis, avascular necrosis of the femoral head, or LCPD.
	Kannu et al. (2011)	Peripheral blood (human)	Two children with *COL2A1* gene mutation/LCPD	Two children had *COL2A1* gene mutations, with clinical manifestations of LCPD.
	Miyamoto et al. (2007)		A family with autosomal dominant hereditary hip disease/LCPD	*COL2A1* gene mutations are more common in LCPD patients, especially in familial cases or cases with bilateral hip involvement.
Factor V Leiden, prothrombin II	Woratanarat et al. (2014)	Peripheral blood (human)	824 children with LCPD, with a control group of 2033 children	Factor V Leiden mutation and prothrombin II polymorphism are significantly associated with LCPD.
Factor V Leiden, prothrombin G20210A, factor VIII	Vosmaer et al. (2010)		169 LCPD patients and 512 controls	The risk of LCPD in men and boys is 2.4 times higher than in women and girls. The risk of LCPD disease increases with the number of coagulation abnormalities in men and boys, but not in women and girls.
Protein C, protein S	Glueck et al. (1994)		8 LCPD patients	Protein C and S deficiency, thrombo, and insufficient fibrinolysis leading to thrombotic venous occlusion of the femur may contribute to the cause of LCPD.
Protein C, factor V Leiden	Glueck et al. (1997)		64 children with LCPD,with a control group of 160 children	In 31 of 64 LCPD patients, the patients with activated protein C and coagulation factor V Leiden were abnormal, and the resistance to activated protein C, prone to thrombosis tendency, may be the pathogenic factor of LCPD。
Protein C, protein S	Eldridge et al. (2001)		57 patients with LCPD and an equal number of controls	The reduction in protein C and S levels can predispose to abnormalities of the coagulation system in a thrombosis-prone state, which may be a risk factor for the interruption of blood supply in patients with LCPD.
Factor V Leiden	Glueck et al. (2007)		A four-generation family/30 members	A tissue factor V mutation was associated with venous and arterial thrombosis in a four-generation family; genetic mutation screening of families of children with LCPD could facilitate screening of relatives with associated thromboabnormal function.
Prothrombin time, FV, FVIII, FIX, and Hcy	Hernández-Zamora et al. (2023)		5 LCPD patients and 50 controls	The shortened prothrombin time and increased FV activity, and elevated FVIII, FIX and Hcy concentrations support the hypothesis that microthrombosis in small caliber vessels may lead to disruption of blood supply and femur osteonecrosis, which are characteristic of LCPD.
LINE-1 promoter methylation	Zheng et al. (2015)		82 LCPD patients and 120 controls	The significant differences in global methylation of DNA from peripheral blood between LCPD patients and matched controls, and aberrant DNA methylation patterns may serve as an epigenetic biomarker for early detection of LCPD.
mRNAs, lncRNAs	Wang et al. (2022)	Periosteum (human)	9 LCPD patients and 6 controls	In the first genome-wide analysis of lncRNA and mRNA expression profiles in the periosteum of LCPD and control patients to screen for altered genes involved in coagulopathy and vascular structural and functional abnormalities, they found 13 differentially expressed lncRNA associated with vascular structural and functional disorders, possibly responsible for blood disruption in LCPD.
miRNA	Huang et al. (2022)	Peripheral blood (human)	3 LCPD patients and 3 controls	The exosomes present in the plasma of LCPD, possibly mediated by miRNA, lead to impaired endothelial cell function and promote osteoclastogenesis.
miR-214	Zhu et al. (2019)	Cartidral tissue and serum of Femoral head (human)	20 LCPD patients and 20 controls	The miR-214 may be expected to be a biomarker or potential target for the diagnosis or treatment of LCPD.
miR-214-3p	Lan et al. (2023)			The microRNA 214 expression was decreased in chondrocytes and serum in LCPD patients, and overexpression of microRNA 214 promoted chondrocyte viability and reduced apoptosis through downregulation of the apoptosis factor Bax.
*IL-6* G-174C and G-597A polymorphisms	Srzentić et al. (2014)	Peripheral blood (human)	37 LCPD patients and 50 controls	The heterozygous carriers of the *IL-6* G-174C/G-597A polymorphism locus had a much lower risk of LCPD than did homozygous carriers.
*IL-6*	Kamiya et al. (2015a)	Hip synovial fluid (human)	28 LCPD patients	In the synovial fluid of the active phase, the levels of several proinflammatory tissue factors, especially the level of *IL-6*.
Endothelial cell markers	Seguin et al. (2008)	Peripheral blood (human)	49 patients with necrosis of the femoral head	The study found a high correlation between abnormal endothelial cell structure or function and necrosis of the femoral head.
Circulating soluble thrombomodulin	Aksoy et al. (2008)		42 LCPD patients and 35 controls	A significant increase in the median values of GFC and TM in LCPD patients, and high levels of soluble TM may be associated with sustained endothelial damage or sustained inflammatory response during the disease process.
Polymorphisms in the *eNOS* gene	Zhao et al. (2016)		80 LCPD patients and 100 controls	This study comparing 27-bp VNTR in exon 4 of nitric oxide synthase (*eNOS*) and G894T in exon 7 showed that the 27-bp VNTR had a higher ab genotype frequency in the LCPD group.
HMGB1, TNF-α, IL-1β, *IL-6*	Kamiya and Kim (2021)	Hip synovial fluid (human)	Patients with LCPD (an unknown number)	The proinflammatory cytokines (HMGB 1), tumor necrosis factor- α (TNF- α), and IL-1β were all increased in the synovial fluid of LCPD patients.

### 2.1 *COL2A1* mutations

Research on *COL2A1* mutations in femoral head ischemic necrosis began with Liu et al. (2005), who conducted a four-generation pedigree analysis of two families with familial femoral head necrosis. They found that familial femoral head necrosis is dominantly inherited and identified a gene mutation in the 12q13 region (Chen et al., 2004), with further sequencing of the *COL2A1* gene exon encoding collagenase II confirming a mutation at codon 1170 (Su et al., 2010). *COL2A1* gene mutations are therefore the cause of familial femoral head ischemic necrosis. LCPD is idiopathic ischemic necrosis of the femoral head in children, and other scholars have also reported the same *COL2A1* gene locus mutations in familial LCPD patients (Kannu et al., 2011; Miyamoto et al., 2007; Woratanarat et al., 2014; Su et al., 2008). Cartilage tissue consists of chondrocytes and cartilage matrix, with collagen II playing a key role in maintaining its homeostasis. *COL2A1* encodes the precursor of the α-1 chain of collagenase II, and the aforementioned mutation results in a substitution of serine for glycine in the Gly-X-Y domain of collagenase II, leading to defects in the synthesis and structure of collagen II (Ibrahim and Little, 2016). This result explains the short stature observed in LCPD patients. This mutation also alters the cartilage matrix structure, reducing its strength. The hip joint is the main weight-bearing joint of the lower limbs, and the combination of load-bearing and reduced biomechanical properties of the epiphysis can lead to vascular occlusion of the epiphysis (Pinheiro et al., 2018). Therefore, a solid theoretical basis for *COL2A1* mutations as a cause of LCPD is identified.

Some scholars have pointed out that although typical LCPD hip joint imaging changes are observed in these cases, it is difficult to distinguish whether these changes are caused by ischemia or by epiphyseal dysplasia. No sporadic cases of LCPD with *COL2A1* mutations have been reported so far (Kim, 2011). *COL2A1* gene mutations may be a cause of familial LCPD, but their role as an independent cause of the disease remains controversial. Chinese scholars recently conducted *COL2A1* mutation testing on a familial LCPD family and identified a novel heterozygous mutation in exon 29 of *COL2A1* (c.1888 G.A, p.Gly630Ser) (Li et al., 2014). This finding suggests the presence of a novel *COL2A1* gene mutation site in LCPD patients, and whether this mutation exists in sporadic cases requires further research with a larger sample size.

### 2.2 Factors associated with coagulation abnormalities

Thrombophilia is a disease caused by abnormal coagulation-related factors (Crowther and Kelton, 2003; Baglin et al., 2010). Vosmaer et al. (2010) found that factor V Leiden, prothrombin G20210A mutations, Protein-S and Protein-C deficiencies, and elevated factor VIII are all associated with an increased risk of LCPD. As the number of abnormal coagulation factors increases, the risk also increases. Thrombophilia can potentially lead to venous thrombosis, which obstructs blood flow from the femoral head epiphysis, resulting in ischemia. Scholars have therefore conducted extensive studies on the relationship between thrombophilia and LCPD.

#### 2.2.1 Protein-C, Protein-S deficiency

Protein C is a vitamin K-dependent plasma zymogen that plays a central role in the inhibition of the blood coagulation cascade. Human protein C is encoded by a gene on chromosome 2ql3-14 and spans approximately 10 kilobases; Protein S is essential for the anticoagulant role of protein C in blood coagulation (Hepner and Karlaftis, 2013; Griffin et al., 2007). Similarly, protein S is a vitamin K-dependent plasma glycoprotein with a molecular weight of approximately 75 kDa, consisting of 635 amino acid residues. Protein S is a potent cofactor in the regulation of the intrinsic pathway by activated protein C (APC) (Dahlbäck, 1991). The strong association between genetic or acquired protein C and protein S deficiencies and increased risk of venous thrombosis suggests that protein C and protein S play important and central roles in controlling the initiation and propagation stages of the coagulation cascade.

As early as 1994, a clinical controlled trial led by Glueck et al. (1994) first reported that among 8 LCPD patients, 3 had Protein-C deficiency and 1 had Protein-S deficiency. After expanding the sample size to 44 cases, they confirmed the presence of anticoagulant factor deficiencies in LCPD, with 19 cases of Protein-C deficiency and 4 cases of Protein-S deficiency. Another study by Glueck found that the factor V Leiden mutation increases resistance to Protein-C, suggesting that this result might explain the deficiency of anticoagulant factors (Glueck et al., 1997). These findings indicate a deficiency of anticoagulant factors in LCPD patients, leading Glueck to propose that thrombophilia is the cause of the disease. Other scholars reached similar conclusions. Eldridge et al. (2001) found that Protein-C and Protein-S levels in the disease group were significantly lower than in the control group. This finding suggests that the deficiency of Protein-C and Protein-S may contribute to the susceptibility to LCPD.

#### 2.2.2 Factor V Leiden mutation

The factor V Leiden mutation is another cause of thrombophilia, and Glueck et al. (1997) initially reported that 8 out of 64 LCPD patients had the factor V Leiden mutation. Another study involving 61 LCPD patients found that the factor V Leiden mutation rate (4.9%) was significantly higher than in the control group (0.7%). Recently, a large-scale Dutch case-control study found that the incidence of LCPD was higher with the factor V Leiden mutation (16/166 vs. 16/509) (Vosmaer et al., 2010). These researchers believe that thrombophilia is a high-risk factor for LCPD. However, scholars from different regions have drawn different conclusions. For instance, Israeli researchers studied 119 LCPD patients and 276 normal children, finding no difference in the factor V Leiden mutation rate between the LCPD group (7/119) and the normal group (13/276) (Kenet et al., 2008).

Given the inconsistent findings, Thaveeratitharm et al. (Miyamoto et al., 2007) conducted a meta-analysis of the current literature to determine whether thrombophilia is related to LCPD. They found that the factor V Leiden mutation increased the risk of LCPD by three times compared with the control group. This study concluded that the factor V Leiden mutation is a risk factor for LCPD. The form of the factor V Leiden mutation involves a substitution of CGA with CAA at position 1691 of the gene (Glueck et al., 1997), and this mutation can be vertically transmitted. A study involving genetic screening of LCPD patients found the factor V Leiden mutation in first- and second-degree relatives, which was associated with thrombotic events within the family (Glueck et al., 2007). Additionally, researchers analyzed blood and plasma samples from 25 LCPD patients and 50 healthy controls, discovering reduced prothrombin time and elevated levels of factor V Leiden, factor VIII, factor IX, and Hcy in LCPD patients (Hernández-Zamora et al., 2023), potentially explaining the coagulation abnormalities in these patients.

In summary, coagulation factor-related abnormalities in blood coagulation may be a cause of LCPD. However, the likelihood of thrombosis in childhood is low, and no reports of coagulation abnormalities in children with LCPD have been documented. A possible explanation is that LCPD may be a hematological disorder, where the mutation potentially affects the coagulation state of the blood system, although the specific mechanism remains unclear.

### 2.3 Epigenetic modifications

Epigenetics suggests that acquired phenotypes can be inherited, and non-genetic changes influence the genotype. Epigenetic regulation includes selective transcriptional and post-transcriptional control, with DNA methylation being one of the modes of selective transcriptional regulation. In normal physiology, DNA methylation regulates the temporal expression of genes during embryogenesis. If methylation and demethylation are imbalanced, this can cause abnormal timing in gene expression. Considering that LCPD commonly occurs in children aged 4–8 and is self-limiting and self-healing (Herring et al., 2004), domestic scholars conducted research on DNA methylation levels in LCPD patients. LINE-1 is a class of non-long terminal repeat retrotransposons, accounting for approximately 18% of the human genome. Given their high frequency presence in the genome, LINE-1 methylation levels can be used as a marker for full DNA methylation levels (Zheng et al., 2015). The study analyzed LINE-1 promoter levels in 82 patients, finding a significant reduction compared with controls. Further subgroup analysis revealed that this difference existed only in male patients (Zheng et al., 2015). This finding correlates with the clinical tendency of LCPD to occur more frequently in men and boys. Furthermore, socioeconomic environment, maternal smoking, and childhood tobacco exposure are risk factors for LCPD (Perry et al., 2017), and these factors are thought to act through epigenetic mechanisms, with DNA methylation being one key regulatory pathway (Ladd-Acosta et al., 2016). Hence, DNA methylation levels could be a critical factor in LCPD pathogenesis.

Non-coding RNA regulation is an important part of epigenetic modifications and is considered a biomarker for clinical diagnosis, with its abnormal expression often involved in the pathogenesis of certain diseases. In LCPD patients, reduced expression of non-coding RNAs (such as lncRNA n335645) may downregulate the expression of related gene mRNAs (ILK, VCL, RRAS, or other genes), leading to vascular structural or functional damage and interruption of blood supply to the femoral head in LCPD patients (Wang et al., 2022). Exosomal miRNA extracted from the plasma of LCPD patients could promote endothelial cell dysfunction and osteoclastogenesis (Huang et al., 2022). *In vitro* experiments found that the expression of microRNA-214 in chondrocytes and serum of LCPD patients was reduced, while overexpression of microRNA-214 promoted chondrocyte viability and reduced apoptosis by downregulating the apoptotic factor Bax. This has potential as a biomarker or therapeutic target for the diagnosis or treatment of LCPD (Zhu et al., 2019; Lan et al., 2023).

In summary, analyzing global DNA methylation levels and non-coding RNA expression in patients provides the first epigenetic explanation for LCPD etiology. This study offers a reference for future etiological research on LCPD. Its limitation lies in only presenting a phenomenon. Whether the reduced DNA methylation and non-coding RNA expression share the same driving factors or originate from the same locus changes, and how these changes affect disease development, remain areas for further research.

### 2.4 Genetic polymorphisms

Changes in DNA bases result in gene mutations, while natural variations in single nucleotides cause DNA sequence polymorphisms. DNA sequence polymorphism refers to the presence of two or more distinct genotypes or alleles, also known as genetic polymorphism. Genetic polymorphisms provide individual susceptibility, and environmental stimuli combined with this susceptibility lead to disease, which we refer to as the phenotype. Srzentić et al. (2014) analyzed *IL-6* and TLR4 gene polymorphisms in 37 children with early-stage LCPD. They found that *IL-6* G-174C/G-597A polymorphisms were in complete linkage disequilibrium, and the proportion of heterozygous carriers of the *IL-6* G-174C/G-597A polymorphism was significantly lower in the disease group than in the control group. The risk of developing LCPD was therefore much lower in heterozygous carriers than in homozygous carriers. Theoretically, homozygous carriers have a genetic susceptibility to elevated *IL-6*. Clinically, Kim et al. (Kamiya et al., 2015a) found elevated levels of various pro-inflammatory factors in the synovial fluid of active LCPD patients, with *IL-6* showing the most significant increase. They also found no TLR4 gene polymorphisms in either group of children, consistent with the theory that LCPD is a form of aseptic inflammation (Srzentić et al., 2014).

Recent studies suggest that endothelial cell structural or functional abnormalities exist in LCPD patients (Seguin et al., 2008; Aksoy et al., 2008; Perry et al., 2012b). Endothelial nitric oxide synthase (*eNOS*) regulates endothelial cell function by synthesizing nitric oxide. Zhao et al. (2016) compared 80 LCPD patients and 100 healthy children, examining the 27-bp VNTR in exon 4 and G894T in exon 7 of the *eNOS* gene. Results showed a higher frequency of the ab genotype for the 27-bp VNTR in the LCPD group. In exon 7, the heterozygous GT genotype for G894T was more frequent in the LCPD group than in the healthy control group. The results suggest that *eNOS* gene polymorphisms may be a risk factor for LCPD. The influence of *IL-6* and *eNOS* gene polymorphisms on LCPD development can be explained by the theory of genetic susceptibility interacting with environmental stimuli (Glueck et al., 2007), though the specific mechanisms require further research.

### 2.5 Other molecules that may affect LCPD

Numerous researchers have investigated biomarkers associated with LCPD patients and discovered elevated expression of relevant molecules in the serum and synovial fluid of these patients. Pro-inflammatory cytokines (HMGB1), tumor necrosis factor-α (TNF-α), and IL-1 have been detected at elevated levels in the synovial fluid of LCPD patients (Kamiya and Kim, 2021). The elevation of these cytokines further upregulates *IL-6* expression, exacerbating the inflammatory response in LCPD patients and leading to synovitis. Compared with healthy controls, LCPD patients have significantly elevated levels of osteocalcin (Wei et al., 2017) and free leptin (Lee et al., 2013) in their serum, which are associated with the staging and severity of LCPD. Undeniably, these molecules are involved in the development of LCPD to some extent, but whether they can serve as independent molecular markers of LCPD requires further research.

## 3 Cell biology

The balance of the femoral head epiphyseal bone depends on the equilibrium between osteoblastic differentiation, osteoclastic differentiation, and bone angiogenesis. Disruption of this balance can lead to disease. In recent years, scholars have studied whether these molecular changes affect cell function and subsequently cause disease. Current research suggests that the development of LCPD may be related to dysfunctions of microvascular endothelial cells and osteoclasts, ischemic damage to chondrocytes, and the recruitment of bone marrow immune cells. The main findings of the included articles are summarized (Table 2).

**TABLE 2:** Main findings of included cohort studies.

Cell Biology	Related molecules	Ref.	Source of specimen	Subjects	Results/Conclusions
Risk factors for abnormal endothelial cell function	Passive smoking/tobacco exposure	Garcia Mata et al. (2000)	—	90 LCPD patients and 183 controls	All 90 patients with LCPD had a history of secondhand smoke exposure, while only 79 of the 183 children in the control group had tobacco exposure. This finding suggests that passive smoking may be a potential or direct risk factor for the development of LCPD.
		Bahmanyar et al. (2008)		852 LCPD patients and 4,432 controls	This retrospective analysis of the tobacco exposure history of 852 LCPD mothers found that maternal exposure to tobacco during pregnancy directly led to a significantly increased risk of LCPD in their offspring.
		Glueck et al. (1998)		39 non-smoking patients with LCPD	24 children out of 39 patients with LCPD had a history of tobacco exposure. Exposure to secondhand smoke in utero and in childhood appears to reduce the activity of plasminogen activator in stimulated tissue, leading to hypofibrinolysis, further leading to the occurrence of thrombotic venous obstruction of the femoral head, leading to LPCD.
		Gunes et al. (2007)		28 maternal smoking newborns and 28 control newborns	The study found significant increase in intimal media thickness in newborns whose mothers smoked, indicating that maternal tobacco exposure during pregnancy increased the risk of cardiovascular disease later in infancy.
		Kallio et al. (2007)	Peripheral blood (human)	402 children aged 8–11 years	The environmental tobacco smoke exposure, in a concentration-dependent manner, impaired vascular endothelial cell function in 11-year-old children.
	*IL-6*	Li et al. (2021)	Peripheral blood (human)	LCPD patients and controls (The number of unknown)	Compared with healthy controls, *IL-6* levels are increased in the blood of LCPD patients, and *IL-6* produces endothelial microparticles (EMPs) by stimulating endothelial cells, thus leading to endothelial dysfunction, which may be a cause of LCPD.
Abnormalities in peripheral blood biomarkers	N/L	Kaymaz et al. (2016)	Peripheral blood (human)	40 LCPD patients and 25 controls	The mean neutrophil to lymphocyte (N/L) ratio was significantly higher in LCPD patients compared with the control group. The N/L ratio may be an important parameter in assessing the natural course of LCPD and can also serve as an independent factor in predicting the prognosis of LCPD patients.
	PLR	Wang et al. (2023)		74 LCPD patients and 60 controls	PLR was a diagnostic value in necrosis and fragmentation stages.
	Serum prolidase activity	Altay et al. (2011)		39 LCPD patients and 40 controls	A comparison of 39 LCPD patients and 40 normal children showed that prolinase activity in serum of LCPD patients was significantly enhanced, and TOS (total oxidative stress) and OSI (oxidative stress index) levels were also significantly increased, while TAC (total antioxidant capacity) showed a downward trend. The results indicate serum prolinase activity as a risk factor for morbidity in LCPD patients.
	E-selectin and P-selectin levels	Vusal Ismayilov (2014)		55 LCPD patients and 30 controls	An upregulation of E-selectin and P-selectin levels in serum samples from 55 LCPD patients. The increased levels of E-selectin and P-selectin, may reflect endothelial activation and/or injury, which in turn leads to the formation of microvascular thrombosis and the development of LCPD.
	Serum osteocalcin	Wei et al. (2017)		20 LCPD patients and 20 controls	Significantly higher serum levels in LCPD patients compared with healthy controls.
	Serum leptin	Lee et al. (2013)		41 LCPD patients and 41controls	The significantly higher free leptin levels in the serum of LCPD patients compared with healthy controls.
Osteoclastic	—	Kim and Su (2002)	Femoral head (piglets)	25 ischemic necrosis in immature pig	In the piglet model of ischemic necrosis, the number of perifibrovascular osteoblasts was small during the repair period, but a large number of osteoclasts were visible, that is, the main repair reaction was osteoclast bone resorption.
		Aruwajoye et al. (2015)		Model of ischemic necrosis of immature pig	The increase in phosphate content in necrotic bone is increased, and the change in apatite composition caused by carbonate substitution, probably due to the increased solubility, plays a key role in the absorption of necrotic bone, increasing the activity of osteoclasts.
	miRNAs	Huang et al. (2022)	Peripheral blood (human)	3 LCPD patients and 3 controls	The miR-3133, miR-4693-3p, miR-4693-5p of plasma-exosomes, miR-141-3p and miR-30a from LCPD patients could activate osteoclasts and promote osteoclastogenesis in vitro, which may account for promoting excessive bone resorption and accelerating femoral head collapse in LCPD patients.
	Diphosphonate	Jamil et al. (2017)	—	100 LCPD patients	The study designed the first randomized controlled trial of bisphosphonates for LCPD to compare standard of care plus bisphosphonates to standard of care between the two treatment groups. Demonstrated that the antiresorptive properties of bisphosphonates are by reducing osteocast activation of LCPD, thereby preventing excessive bone resorption of the femoral head leading to collapse.
		Aya-ay et al. (2007)	Femoral head (piglets)	27 ischemic necrosis in immature pig	Intraosseous administration (bisphosphonate) inhibited the overactivation of osteoclasts, thus effectively preserving the head structure of the femoral head and preventing its deformation.
		Kim et al. (2005)		24 ischemic necrosis in immature pig	Ibandronate preserves the trabecular structure of the epiphysis and prevents femoral head deformity during the early stages of ischemic necrosis repair in theischemic necrosis in the immature pig model.
	BMP-2+diphosphonate	Kim et al. (2014)		17 ischemic necrosis in immature pig	BMP-2 in combination with bisphosphonates significantly reduced bone resorption and increased new bone formation in ischemic necrosis in the immature pig, in which bisphosphonates were achieved by inhibition of osteocast activation.
	RANKL inhibitor	Kim et al. (2006)		18 ischemic necrosis in immature pig	This study is the first to demonstrate that the use of RANKL inhibitors effectively reduces bone resorption and permanent femoral head deformity (FHD) after ischemic osteonecrosis in ischemic necrosis in the immature pig.
Macrophages	TLR4	Adapala et al. (2016)	Bone marrow (piglets)	Ischemic necrosis in immature pig	Necrotic epiphysis then stimulate the M1 pro-inflammatory response of macrophages, which partly aggravates the progression of LCPD.
	miR-214-3p	Lan et al. (2023)	Bone chondrogenic tissue and peripheral blood of LCPD patients	Patients with LCPD (an unknown number)	In vitro, chondrocell exosomes overexpressing miR-214-3p significantly promoted M2 type macrophage polarization and angiogenesis and accelerated the repair of ischemic necrosis of the femoral epiphysis, thus alleviating LCPD.
Epiphyseal cartilage, chondrocytes	Lipid droplet	Kitoh et al. (2008)	Cartilage tissue of iliac crest (human)	11 children with LCPD underwent iliac osteochondral osteotomy and 10 children underwent Salter osteotomy	Higher lipid droplets and in cytoplasmic contents rich in fibrils in chondrocytes. These changes may play key roles in the degenerated matrix and contribute to the vulnerability of the cartilage tissue. Abnormalities in the metabolic function of chondrocytes may be associated to the pathogenesis of some LCPD patients.
	VEGF	Kim et al. (2004a)	Femoral head (piglets)	Ischemic necrosis in immature pig	Most of the epiphyseal chondrocytes of piglets developed necrosis after ischemia, leading to the cessation of ossification in the epiphyseal cartilage and necrotic collapse of the femoral head. VEGF protein and mRNA expression were increased in the epiphyseal cartilage after infarction. This may play a key role in promoting vascular invasion and granulation tissue formation in areas of epiphyseal cartilage necrosis, and this process may be an important step in promoting necrotic cartilage resorption and restoring endochondral ossification, thus leading to further growth and development of the femoral head.
	HIF-1 and *IL-6*	Yamaguchi et al. (2016)			The induction of ischemic osteonecrosis in immature pigs produces *IL-6* in articular cartilage through a hif-1-dependent pathway, and *IL-6* produced by hypoxic articular chondrocytes further stimulates the inflammatory cytokine response in synoviocytes, leading to the development of hip synovitis after osteonecrosis.
	HIF-1α and Sox9	Zhang et al. (2011)			HIF-1 α and Sox 9 were upregulated in deep chondrocytes stimulated by ischemic hypoxia, so the HIF-1 α of Sox 9 activity may have a cartilage protective effect after femoral head ischemia, which then promoted epiphyseal revascularization and recovery of endochondral ossification.
	—	Rush et al. (1988)	—	20 LCPD patients	MRI imaging provides a means to assess the condition of the acetabular and epiphyseal cartilage in patients with LCPD, better assessing the containment of the femoral head, acetabular and femoral articular surfaces, and soft tissue abnormalities in the articular capsule.
		Mastantuono et al. (1997)		9 LCPD patients	MRI accurately defined the anatomical profile of the normal hip. MRI can locate abnormal areas early; it can show changes throughout the cartilage of the femoral head and the epiphyseal nucleus.
		Pienkowski et al. (2009)		10 LCPD patients	The significant advantages of MRI technique in objectively assessing cartilage morphology in LCPD patients and was helpful in future diagnostic work. It enables three-dimensional reconstruction of the femur and acetabulum and quantitative analysis of therapeutic effects.
		Johnson et al. (2018)		Ischemic necrosis in the immature pig	T1 ρ and T2 mapping showed high sensitivity for MRI imaging to detect secondary ossification center (SOC) and femoral seal cartilage damage caused by 48-hour ischemia in piglets.
		Johnson et al. (2022)			
		Armstrong et al. (2022)			
		Jones et al. (2022)		12 cured LCPD patients and 15 healthy controls	Cartilage damage in LCPD began to manifest during adolescence. The T1 ρ imaging technique is able to detect the early changes in cartilage associated with LCPD.

### 3.1 Abnormal micro endothelial cell function

The physiological vascular changes in the femoral head of children have particular characteristics. In children aged 1–3 years, the femoral head epiphysis is supplied by the lateral circumflex femoral artery and the medial circumflex femoral artery. Between ages 4–7, the lateral circumflex artery to the epiphysis closes, and after age 7, the ligamentum teres artery grows in, the lateral circumflex artery reopens, and blood supply to the femoral head epiphysis is restored (Berthaume et al., 2016). LCPD commonly occurs in children aged 4–8 years (Herring et al., 2004), aligning with this physiological timeline. Theoretically, if the structure of the medial circumflex artery is abnormal or if angiogenesis is inhibited, leading to delayed reopening, disease may occur. Endothelial cells play an important role in angiogenesis, leading scholars to suggest that children with LCPD may have structural or functional abnormalities in their vascular endothelium (Seguin et al., 2008; Aksoy et al., 2008; Perry et al., 2012b; Hailer et al., 2010). This hypothesis is supported by epidemiological and clinical examinations.

### 3.2 Risk factors for microvascular endothelial cell dysfunction

The mainstream view holds that maternal passive smoking and/or postnatal infant tobacco exposure are closely related to the onset of LCPD. In 2000, Garcia Mata et al. (2000) reported that all 90 LCPD patients in the disease group had a history of secondhand smoke exposure, whereas only 79 of 183 control group children had tobacco exposure. Although no correlation was found between tobacco exposure and Catterall staging or Stulberg classification, the risk of LCPD in children exposed to passive smoking was more than five times higher than in normal children. In a retrospective analysis by Swedish scholars of 852 LCPD patients, maternal tobacco exposure during pregnancy was found to significantly increase the risk of LCPD in offspring (Bahmanyar et al., 2008). Subsequent studies by other scholars supported this view, with Daniel et al. (2012) finding that environmental tobacco exposure and smoke from indoor wood burning also increased the risk of the disease. Recently, Perry et al. (2017) suggested that environmental tobacco exposure and maternal tobacco exposure during pregnancy are risk factors for LCPD, potentially explaining the association between socioeconomic status and LCPD. Tobacco exposure is a risk factor for LCPD, and how it influences the occurrence of LCPD has attracted the interest of scholars. Glueck et al. (1998) reported that out of 39 LCPD patients, 24 children had a history of tobacco exposure, with 48% having low tissue plasminogen activator activity, significantly higher than the normal group. Thus, Glueck suggested that tobacco exposure leads to hypofibrinolysis, potentially further causing thrombotic venous occlusion in the femoral head, resulting in LCPD. Our research group previously found that serum endothelial microparticles (Li et al., 2021) and miRNA in plasma exosomes (Huang et al., 2022) from LCPD patients can promote endothelial dysfunction and inhibit angiogenesis in LCPD patients. Previous studies have confirmed that maternal tobacco exposure during pregnancy increases the risk of cardiovascular disease in infants, and tobacco exposure elevates oxidative stress levels in the body, damaging endothelial cell function in a dose-dependent manner (Gunes et al., 2007; Kallio et al., 2007). Some researchers therefore speculate that LCPD may not be caused by coagulation system abnormalities, but by vascular structural or functional issues, particularly endothelial dysfunction.

Some scholars have suggested that hip joint synovitis in LCPD is a chronic process and may be involved in the onset and progression of the disease. During the repair phase of femoral head ischemic necrosis, excessive resorption of necrotic bone, delayed new bone formation, and replacement of necrotic bone with fibrous vascular tissue occur. These processes persist over time. Using gadolinium-enhanced MRI, Kim et al. (Neal et al., 2015) detected increased synovitis signals in the affected hip, and these signals correlated with Waldenström staging. These findings suggest that synovitis is a chronic condition. To further explore the nature of hip synovitis, they performed cytokine quantification on joint fluid from active LCPD patients and found significant elevations in pro-inflammatory cytokines, with *IL-6* being the most notably increased (Kamiya et al., 2015a). *IL-6*, as a pro-inflammatory cytokine, can directly damage endothelial cells and induce oxidative stress, leading to endothelial dysfunction, aligning with the findings of our research group (Li et al., 2021; Liu et al., 2024). Furthermore, Kim et al. (Kuroyanagi et al., 2018; Kamiya et al., 2019) demonstrated that *IL-6* knockout mice showed restored angiogenesis following epiphyseal ischemic necrosis. The rise in pro-inflammatory cytokines in hip synovitis therefore acts as a risk factor for microvascular endothelial cell damage, potentially resulting in suppressed angiogenesis.

### 3.3 Abnormal peripheral blood biomarkers

Vascular endothelial cells are important endocrine organs that regulate vascular tone, platelet aggregation, coagulation, and fibrinolysis. Biomarkers in the blood can reflect endothelial cell function, and in recent years, scholars have focused on how these biomarker levels present in LCPD patients. Researchers analyzing blood biomarkers in LCPD patients found that the neutrophil-to-lymphocyte ratio (N/L) (Kaymaz et al., 2016) and platelet-to-lymphocyte ratio (PLR) (Wang et al., 2023) were significantly higher in the LCPD group than in the control group, with the increase in N/L being most pronounced in the good prognosis Herring A/B group. These blood markers can reflect the body’s inflammation levels and are important for the diagnosis and treatment of LCPD. Reactive oxygen species (ROS) are present in the normal body, but excessive ROS can lead to endothelial cell damage. Altay et al. (2011) compared 39 LCPD patients with 40 normal children in terms of serum proline enzyme activity, total oxidant status (TOS), total antioxidant capacity (TAC), and oxidative stress index (OSI). They found that serum proline enzyme activity, TOS, and OSI were significantly elevated, while TAC was reduced in LCPD patients. These results suggest that elevated serum proline enzyme activity is a risk factor for LCPD. E-selectin and P-selectin shed from endothelial cells during activation or apoptosis, entering the bloodstream, and can reflect the functional status of microvascular endothelial cells. Vusal Ismayilov (2014) measured serum levels of E-selectin and P-selectin in 85 LCPD patients and found both to be upregulated in LCPD patients. Elevated E-selectin and P-selectin levels stimulate platelet and endothelial cell activation, leading to microvascular thrombosis and the development of LCPD. Additionally, endothelial nitric oxide synthase, the primary enzyme for nitric oxide production, exhibits increased gene polymorphisms in LCPD patients, elevating LCPD risk and potentially affecting endothelial cell relaxation function (Zhao et al., 2016). Despite the limited number of cases, these abnormal serum biomarkers reflect endothelial dysfunction in LCPD patients.

### 3.4 Microvascular endothelial cell function tests

The interruption of blood supply to the femoral head epiphysis is an established mechanism of LCPD, but research has largely focused on extravascular factors, with little emphasis on the functional state of the vessels themselves. Swedish scholars conducted a retrospective analysis of 3,141 LCPD patients and performed regression analysis on the relationship with cardiovascular disease. They found that compared with non-LCPD patients, the risk ratio for cardiovascular disease was 1.70, and the risk of hypertension was statistically significantly higher (Hailer et al., 2010; Mörlin and Hailer, 2021). This result suggests that vascular dysfunction may be prevalent among LCPD patients. To further investigate the structural and functional state of microvascular endothelial cells in LCPD, the research team applied flow-mediated dilation (FMD) to directly assess endothelial function and recorded changes in arterial blood flow under ischemic stimuli. They found that the microvascular diameter supplying LCPD patients was smaller and blood flow velocity slower compared with the normal group, while the large vessels remained in a normal state (Perry et al., 2012b). The significance of this study lies in its direct revelation of structural or functional abnormalities in the microvascular endothelial cells of LCPD patients, providing direct evidence of endothelial dysfunction in LCPD.

### 3.5 Abnormal activation of osteoclasts

The balance of bone remodeling depends on the balance between the number and function of osteoclasts, bone angiogenesis, and osteoblasts. Once this balance is disrupted, it can lead to disease. Clinical studies on the role of bone cells in the pathophysiology of LCPD are scarce, with most insights derived from basic research in animal models. The femoral head epiphysis of piglets has a similar anatomical structure to humans, and other scholars have conducted extensive studies using the piglet LCPD model (Kim and Su, 2002; Kim et al., 2001; Pringle et al., 2004; Koob et al., 2007; Kim et al., 2009).

In the early stages after ischemia, diffuse cell necrosis, apoptosis, and disruption of bone marrow stroma are observed in the femoral head epiphysis, followed by the formation of characteristic necrotic cavities, with osteoblast loss in the trabeculae (Kim et al., 2001). In adults, the repair phase of femoral head ischemic necrosis occurs through “creeping substitution,” where bone marrow mesenchymal stem cells differentiate into osteoblasts to repair necrotic bone. The repair process in LCPD differs from this process. During the repair phase, arteries from the femoral neck regenerate and extend into the secondary ossification center from the lateral side of the epiphyseal cartilage, restoring blood supply to the femoral head epiphysis (Kim et al., 2016). Osteoblasts around the fibrous vasculature are fewer, but a large number of osteoclasts are observed (Kim and Su, 2002). In early LCPD, hip joint synovitis is an aseptic inflammation, so why are osteoclasts activated? The current view is that the increase in necrotic bone and the chronic inflammatory state of the hip joint are two causes of this activation. Kim et al. (Aruwajoye et al., 2015) found that phosphate levels in necrotic bone were elevated, and phosphate replaced hydroxyapatite, increasing osteoclast activity. Elevated pro-inflammatory cytokines in the hip joint synovial fluid of patients can also activate osteoclasts. Our research group previously found that plasma exosomal miR-3133, miR-4693-3p, miR-4693-5p, miR-141-3p, and miR-30a from LCPD patients could activate osteoclasts and promote osteoclastogenesis *in vitro*. These effects may contribute to excessive bone resorption and accelerated femoral head collapse in LCPD patients (Huang et al., 2022).

TLRs are important immune response receptors in the human body and can recognize ligands through two pathways: pathogen-associated molecular patterns (PAMPs) and damage-associated molecular patterns (DAMPs). Necrotic tissue or damaged cells release cytokines that are recognized by TLRs, activating downstream pathways, which is the mechanism of DAMPs. TLR4 belongs to the TLR family and is primarily expressed on the surface of monocytes and macrophages. Monocytes and macrophages can either promote or suppress inflammation depending on the environment, and pro-inflammatory monocytes can differentiate into osteoclasts, playing a role in bone resorption. Scholars found that in necrotic bone from the femoral head epiphysis in LCPD, phosphate replaces hydroxyapatite, leading to elevated phosphate levels (Aruwajoye et al., 2015). They concluded that necrotic bone is the source of high phosphate levels, which induces monocyte differentiation into osteoclasts through the DAMPs pathway, leading to enhanced bone resorption in the femoral head epiphysis of LCPD patients.

The repair process in LCPD is also accompanied by chronic inflammation. Kim et al. (Adapala et al., 2016) found that necrotic bone could activate the expression of pro-inflammatory cytokines. Through the DAMPs pathway, TLR4 activates monocytes, which then exhibit the pro-inflammatory M1 phenotype. Furthermore, cytokine quantification of joint fluid from active LCPD patients revealed elevated pro-inflammatory cytokines compared with normal controls, with the most significant difference seen in *IL-6* levels (Kamiya et al., 2015a). *IL-6* is part of the tumor necrosis factor (TNF) superfamily. Previous studies have shown that this family can induce osteoclast differentiation, with TNF-α and RANKL being key cytokines involved in osteoclast differentiation (Boyle et al., 2003). To determine whether *IL-6* has a similar function in LCPD, Kim et al. constructed *IL-6* knockout mice and found that osteoclast differentiation was inhibited in *IL-6* knockout mice, femoral head epiphyseal ischemic necrosis deformities were alleviated, and femoral head bone mass increased (Kuroyanagi et al., 2018; Kamiya et al., 2019; Kim et al., 2018).

Osteoclasts are the only known cells responsible for bone resorption in the human body, and this discussion shows that their abnormal activation plays a role in the development of LCPD and contributes to deformity during healing. Studying osteoclasts can provide guidance for clinical treatment (Jamil et al., 2017). For example, the use of bisphosphonates (Aya-ay et al., 2007; Kim et al., 2005) combined with BMP2 (Kim et al., 2014) or RANKL (Kim et al., 2006) to treat ischemic bone necrosis improved femoral head deformities, proving the important role of osteoclast bone resorption in the development of femoral head deformities. Thus, from an etiological perspective and for guiding clinical treatment, the role of osteoclasts in LCPD is worthy of attention.

### 3.6 H-vascular endothelial cells and immune cells

Unlike adult femoral head necrosis, LCPD is a self-limiting disease, and children with LCPD can heal without treatment, which may be related to the higher abundance of H-type vessels in young populations. H-type vessels are a new subtype of capillaries discovered by Kusumbe et al. (2014) in 2014 in the skeletal system of mice. These vessels couple angiogenesis and osteogenesis and play an important role in promoting bone repair. In human bone sections, the abundance of H-type vessels decreases with age, consistent with findings in mouse skeletal systems (Wang et al., 2017). Compared with other types of capillaries, H-type vessels express high levels of CD31 and endomucin. Endothelial cells in H-type vessels interact with bone cells (such as osteoblasts, osteoclasts, and chondrocytes) through cytokines or signaling pathways to maintain bone growth and homeostasis, playing a crucial role in bone diseases such as osteoporosis, osteoarthritis, and osteonecrosis (Liu et al., 2023; Qin et al., 2023; Xu et al., 2024; Ma et al., 2021).

With the introduction of the concept of osteoimmunology, the immune-inflammatory mechanisms between bone and the immune system have become a major focus in the study of bone metabolism-related diseases. In LCPD, necrotic epiphyses activate M1 pro-inflammatory responses in macrophages through Toll-like receptor 4, exacerbating the progression of LCPD to some extent (Adapala et al., 2016). However, *in vitro*, exosomes from chondrocytes overexpressing miR-214-3p can significantly promote M2 macrophage polarization and angiogenesis, thereby alleviating LCPD (Lan et al., 2023; Yu et al., 2023). In the early stages of LCPD bone necrosis, scholars have found that neutrophils and macrophages are recruited to the ischemic necrotic bone area, and their levels remain high throughout the bone repair phase (Phipps et al., 2016). This evidence is the most direct of immune cell involvement in bone necrosis, but how these immune cells contribute to the inflammatory response of bone necrosis and later promote bone repair is still unknown. Other immune cells in the bone marrow microenvironment (such as plasma cells, T cells, and B cells) have been shown to play important roles in bone metabolism-related diseases (Miron et al., 2024; Schlundt et al., 2023; Ma et al., 2022), but no studies on their role in LCPD have been reported. Therefore, exploring the etiology of LCPD from the perspective of osteoimmunity and H-type vessels, and even unraveling the mechanism of self-repair in this disease, may offer more possibilities for future clinical drug development.

### 3.7 Chondrocytes and epiphyseal cartilage

The necrotic collapse of the femoral head in LCPD patients is related to epiphyseal cartilage degeneration. Ischemic injury to the epiphyseal cartilage leads to abnormal chondrocyte metabolism, resulting in disordered matrix calcification. Metabolic damage to ischemic chondrocytes in the epiphysis leads to fragility of the femoral epiphysis, which may be related to the development of LCPD in some patients (Kitoh et al., 2008). In a study on ischemia in immature piglets, Kim et al. (2004a) found that most epiphyseal chondrocytes underwent necrosis after ischemia, resulting in the cessation of endochondral ossification and necrotic collapse of the femoral head. Subsequently, deeper chondrocytes, under the stimulus of ischemia and hypoxia, secrete relevant cytokines that promote the reconstruction of epiphyseal blood flow and the restoration of endochondral ossification (Zhang et al., 2011). In the early stages of LCPD, metabolic dysfunction of superficial chondrocytes in the joint leads to necrotic collapse of the epiphyseal cartilage, after which the surviving deep chondrocytes initiate re-ossification and revascularization of the epiphysis. The KIM team also found that *IL-6* levels are increased in the hip synovial fluid of LCPD patients, and articular chondrocytes are the main source of increased *IL-6* levels, potentially contributing to the occurrence of hip synovitis in LCPD patients (Yamaguchi et al., 2016). Depression of *IL-6* production in articular chondrocytes by using the *IL-6* receptor inhibitor (tocilizumab) (Kamiya et al., 2019) or knockout of the *IL-6* gene (Kuroyanagi et al., 2018) significantly promotes cartilage synthesis, bone formation, and revascularization after ischemic osteonecrosis. To more accurately identify the pathological changes in the femoral epiphysis in LCPD, imaging techniques can help diagnose the early pathological changes of the disease. As early as 1988, Rush et al. (1988) discovered through MRI imaging that it is possible to better assess the ischemic damage to the acetabulum and epiphyseal cartilage in LCPD patients. T1-weighted MRI images can clearly distinguish between the stages of vascular necrosis and fragmentation, and cartilage deformities on MRI are useful for differentiating these stages (Kumasaka et al., 1991). Imaging scans of the hip joints of nine children with LCPD at different stages revealed that MRI could identify abnormal areas earlier than conventional X-rays; it also showed changes in the evolution of the entire femoral head cartilage and epiphyseal nucleus, potentially distinguishing the evolution and occult patterns of osteochondropathy (Mastantuono et al., 1997; Pienkowski et al., 2009; Johnson et al., 2018). This is essential for studying the biomechanics of hip osteochondropathy and developing treatment plans. In adulthood, LCPD patients continue to experience cartilage degeneration of the femoral head, leaving varying degrees of femoral head deformities and even leading to early-onset hip osteoarthritis. However, with new MRI techniques, T1ρ and T2 mapping can sensitively detect ischemic damage to the femoral head SOC and epiphyseal cartilage within 48 h of ischemia induction (Johnson et al., 2022; Armstrong et al., 2022). T1ρ sequence scanning of the hip joint during the healing phase of LCPD can accurately detect early pathological changes in cartilage degeneration in LCPD patients, allowing for early prevention and therapeutic intervention (Jones et al., 2022). These techniques can be used clinically to assess the damage and repair of epiphyseal cartilage, providing better analysis of the ischemic pathological changes in LCPD.

In 22 LCPD patients, scholars obtained cylindrical biopsy specimens from the femoral metaphysis, and histopathological examination showed fat necrosis, vascular proliferation, and focal fibrosis, indicating previous ischemic episodes (Eckerwall et al., 1997). However, bone specimens from LCPD patients are difficult to obtain clinically, so using animal models is an important tool for studying the pathogenesis of Perthes disease and conducting *in vivo* experiments to more accurately observe the pathological changes of LCPD at the histological level. Current research uses animal models to simulate the pathological changes of LCPD. The main animals used include piglets, rabbits, rats, mice, and dogs, each with its own advantages and disadvantages. The advantage of the piglet LCPD model is that it better demonstrates the pathological changes of the femoral head in LCPD patients at the tissue level. Whether through imaging or histological staining, the model accurately presents the necrosis and repair process of LCPD, as well as the pathological changes in the acetabulum (Johnson et al., 2018; Kim et al., 2004b; Miashiro et al., 2023). However, piglets are large, have strict feeding requirements, high experimental costs, and are difficult to model, which are challenges many scholars face. Canine animal models share similar characteristics. Medium-sized animal models like rats (Boss et al., 2003; Little et al., 2005) and rabbits (Kamegaya et al., 1990) are easier to model, less expensive, and simpler to observe. Mouse models (Kamiya et al., 2015b) are better suited for subsequent genetic studies, aiding in the exploration of molecular or gene mutations in LCPD patients, as mentioned earlier in the article. In summary, clinical samples of LCPD are difficult to obtain, and animal model research helps us observe the histopathological changes of LCPD, offering the potential for cellular biotherapy and molecular genetic manipulation, providing more reliable theoretical support for elucidating the etiology and improving the diagnosis and treatment of LCPD.

## 4 Conclusion and outlook

Molecular-level changes create a predisposition to disruption of blood supply to the femoral head. For example, *COL2A1* mutations weaken the strength of the epiphyseal cartilage matrix, mutations in Factor V Leiden, and deficiencies in Protein-C and Protein-S contribute to thrombophilia, while nitric oxide synthase and *IL-6* gene polymorphisms potentially affect endothelial cell function. Pro-inflammatory factors in joint fluid, serum-free osteocalcin, and leptin levels all influence the progression of LCPD to some extent. These susceptibilities, when triggered by environmental factors, lead to the development of LCPD. This finding aligns with Kim’s (Glueck et al., 2007) view that the mechanism of LCPD involves genetic susceptibility combined with environmental factors. Molecular-level changes further impact cellular functions. Increasing reports suggest that structural or functional abnormalities of microvascular endothelial cells are common in LCPD patients, but whether endothelial dysfunction is the cause of LCPD or an intermediate consequence of other risk factors remains to be further investigated. Osteoclasts, the only cells responsible for bone resorption, are activated under LCPD pathological conditions, leading to increased bone resorption. This finding explains clinical manifestations like femoral head deformity and decreased bone density, but the molecular mechanisms require further exploration.

As research on LCPD progresses, the immune response mechanism between bones and immune cells is found to play an important role in LCPD. M1 polarization of bone macrophages promotes the inflammatory response in LCPD, while M2 polarization supports vascular regeneration in LCPD. Other immune cells in the bone marrow have also been shown to play important roles in bone metabolism and repair, but the exact molecular mechanisms in LCPD remain to be elucidated.

Currently, extensive research has been conducted on the development of LCPD animal models. Large animals (such as piglets and puppies) and medium-sized animals (such as rats and rabbits) are more suitable for observing pathological changes in the femoral head, joint cartilage, and epiphyseal cartilage, as well as vascular and connective tissue regeneration at the histopathological level. Mouse models are better suited for molecular-level studies. In juvenile mouse models of femoral epiphyseal ischemic necrosis, repair of epiphyseal necrosis is accompanied by vascular reconstruction and bone repair (Miashiro et al., 2023). This repair may occur through signaling pathways that stimulate endothelial cells to secrete angiogenic and osteogenic factors, consistent with findings by Kim et al. (Boyle et al., 2003; Kim et al., 2018) in piglet models. The self-repair mechanism of LCPD may be related to the osteogenesis-angiogenesis coupling function of H-type vessels. H-type vessels, which are bone-specific vessels that couple osteogenesis and angiogenesis, promote bone repair and vascular regeneration in bone-related diseases, and may become a potential target for treating LCPD. Overall, while the cause of LCPD remains unclear, advancements in molecular and cellular biology offer new perspectives for basic research into the disease.

## Abbreviations

LCPD, Legg-Calvé-Perthes disease; *COL2A1*, Collagen Type II Alpha 1 Chain; IGF-1, Insulin-like growth factor 1; *IL-6*, Interleukin-6; GFC, Global fibrinolytic capacity; TM, Thrombomodulin; VNTR, Variable Number of Tandem Repeats; *eNOS*, Endothelial nitric oxide synthase; HMGB1, Pro-inflammatory cytokines; TNF-α, Tumor necrosis factor-α; IL-1β, Interleukin-1β; LINE-1, Long Interspersed Nuclear Elements 1; TLR4, Toll-like receptors 4; TOS, Total oxidant status; TAC, Total antioxidant capacity; OSI, Oxidative stress index; VEGF, Vascular endothelial growth factor; HIF-1, Hypoxia-inducible factor-1; Sox9, SRY-box transcription factor 9; ROS, Reactive oxygen species.

## References

[B1] Adapala N. S. Yamaguchi R. Phipps M. Aruwajoye O. Kim H. K. W. (2016). Necrotic bone stimulates proinflammatory responses in macrophages through the activation of toll-like receptor 4. Am. J. Pathol. 186, 2987–2999. 10.1016/j.ajpath.2016.06.024 27648614

[B2] Aksoy M. C. Aksoy D. Y. Haznedaroglu I. C. Sayinalp N. Kirazli S. Alpaslan M. (2008). Thrombomodulin and GFC levels in Legg-Calve-Perthes disease. Hematology 13, 324–328. 10.1179/102453308X343509 19055859

[B3] Altay M. A. Erturk C. Aksoy N. Taskin A. Bilge A. Celik H. (2011). Serum prolidase activity and oxidative-antioxidative status in Legg-Calve-Perthes disease. J. Pediatr. Orthop. B 20, 222–226. 10.1097/BPB.0b013e32834493df 21304409

[B4] Armstrong A. R. Bhave S. Buko E. O. Chase K. L. Tóth F. Carlson C. S. (2022). Quantitative T2 and T1ρ mapping are sensitive to ischemic injury to the epiphyseal cartilage in an *in vivo* piglet model of Legg-Calvé-Perthes disease. Osteoarthr. Cartil. 30, 1244–1253. 10.1016/j.joca.2022.05.009 PMC9378508 35644462

[B5] Aruwajoye O. O. Kim H. K. Aswath P. B. (2015). Bone apatite composition of necrotic trabecular bone in the femoral head of immature piglets. Calcif. Tissue Int. 96, 324–334. 10.1007/s00223-015-9959-7 25660159

[B6] Aya-ay J. Athavale S. Morgan-Bagley S. Bian H. Bauss F. Kim H. K. (2007). Retention, distribution, and effects of intraosseously administered ibandronate in the infarcted femoral head. J. Bone Min. Res. 22, 93–100. 10.1359/jbmr.060817 17166092

[B7] Baglin T. Gray E. Greaves M. Hunt B. J. Keeling D. Machin S. (2010). Clinical guidelines for testing for heritable thrombophilia. Br. J. Haematol. 149, 209–220. 10.1111/j.1365-2141.2009.08022.x 20128794

[B8] Bahmanyar S. Montgomery S. M. Weiss R. J. Ekbom A. (2008). Maternal smoking during pregnancy, other prenatal and perinatal factors, and the risk of Legg-Calve-Perthes disease. Pediatrics 122, e459–e464. 10.1542/peds.2008-0307 18625663

[B9] Baş V. N. Uytun S. Vurdem Ü E. Torun Y. A. (2015). Hypopituitarism and Legg-Calve-Perthes disease related to difficult delivery. Korean J. Pediatr. 58, 270–273. 10.3345/kjp.2015.58.7.270 26300943 PMC4543188

[B10] Berthaume M. A. Perry D. C. Dobson C. A. Witzel U. Clarke N. M. Fagan M. J. (2016). Skeletal immaturity, rostral sparing, and disparate hip morphologies as biomechanical causes for Legg-Calvé-Perthes' disease. Clin. Anat. 29, 759–772. 10.1002/ca.22690 26780125

[B11] Boss J. H. Misselevich I. Peskin B. Zinman C. Levin D. Norman D. (2003). Postosteonecrotic osteoarthritis-like disorder of the femoral head of rats. J. Comp. Pathol. 129, 235–239. 10.1016/s0021-9975(03)00031-8 12921731

[B12] Boyle W. J. Simonet W. S. Lacey D. L. (2003). Osteoclast differentiation and activation. Nature 423, 337–342. 10.1038/nature01658 12748652

[B13] Calve J. (2006). On a particular form of pseudo-coxalgia associated with a characteristic deformity of the upper end of the femur. 1910. Clin. Orthop. Relat. Res. 451, 14–16. 10.1097/01.blo.0000238799.05338.5a 17038924

[B14] Catterall A. (1971). The natural history of Perthes' disease. J. Bone Jt. Surg. Br. 53, 37–53. 10.1302/0301-620x.53b1.37 5578764

[B15] Chen W.-M. Liu Y.-F. Lin M.-W. Chen I.-C. Lin P.-Y. Lin G.-L. Jou Y.-S. (2004). Autosomal dominant avascular necrosis of femoral head in two Taiwanese pedigrees and linkage to chromosome 12q13. Am. J. Hum. Genet. 75, 310–317. 10.1086/422702 15179599 PMC1216065

[B16] Crowther M. A. Kelton J. G. (2003). Congenital thrombophilic states associated with venous thrombosis: a qualitative overview and proposed classification system. Ann. Intern Med. 138, 128–134. 10.7326/0003-4819-138-2-200301210-00014 12529095

[B17] Dahlbäck B. (1991). Protein S and C4b-binding protein: components involved in the regulation of the protein C anticoagulant system. Thromb. Haemost. 66, 049–061. 10.1055/s-0038-1646373 1833851

[B18] Daniel A. B. Shah H. Kamath A. Guddettu V. Joseph B. (2012). Environmental tobacco and wood smoke increase the risk of Legg-Calve-Perthes disease. Clin. Orthop. Relat. Res. 470, 2369–2375. 10.1007/s11999-011-2180-8 22090357 PMC3830089

[B19] Eckerwall G. Hochbergs P. Simesen K. Willén H. Egund N. Wingstrand H. (1997). Metaphyseal histology and magnetic resonance imaging in Legg-Calvé-Perthes disease. J. Pediatr. Orthop. 17, 659–662. 10.1097/00004694-199709000-00016 9592007

[B20] Eldridge J. Dilley A. Austin H. M E.L.-J. Wolstein L. Doris J. (2001). The role of protein C, protein S, and resistance to activated protein C in Legg-Perthes disease. Pediatrics 107, 1329–1334. 10.1542/peds.107.6.1329 11389252

[B21] Gao H. Huang Z. Jia Z. Ye H. Fu F. Song M. (2020). Influence of passive smoking on the onset of Legg-Calvè-Perthes disease: a systematic review and meta-analysis. J. Pediatr. Orthop. B 29, 556–566. 10.1097/bpb.0000000000000725 32141957

[B22] Garcia Mata S. Ardanaz Aicua E. Hidalgo Ovejero A. Martinez Grande M. (2000). Legg-Calve-Perthes disease and passive smoking. J. Pediatr. Orthop. 20, 326–330. 10.1097/01241398-200005000-00011 10823599

[B23] Glueck C. J. Brandt G. Gruppo R. Crawford A. Roy D. Tracy T. (1997). Resistance to activated protein C and Legg-Perthes disease. Clin. Orthop. Relat. Res. 338, 139–152. 10.1097/00003086-199705000-00021 9170375

[B24] Glueck C. J. Freiberg R. A. Crawford A. Gruppo R. Roy D. Tracy T. (1998). Secondhand smoke, hypofibrinolysis, and Legg-Perthes disease. Clin. Orthop. Relat. Res. 352, 159–167. 10.1097/00003086-199807000-00019 9678044

[B25] Glueck C. J. Glueck H. I. Greenfield D. Freiberg R. Kahn A. Hamer T. (1994). Protein C and S deficiency, thrombophilia, and hypofibrinolysis: pathophysiologic causes of Legg-Perthes disease. Pediatr. Res. 35, 383–388. 10.1203/00006450-199404000-00001 8047373

[B26] Glueck C. J. Tracy T. Wang P. (2007). Legg-Calve-Perthes disease, venous and arterial thrombi, and the factor V Leiden mutation in a four-generation kindred. J. Pediatr. Orthop. 27, 834–837. 10.1097/BPO.0b013e31815584bf 17878795

[B27] Griffin J. H. Fernández J. A. Gale A. J. Mosnier L. O. (2007). Activated protein C. J. Thromb. Haemost. 5 (Suppl. 1), 73–80. 10.1111/j.1538-7836.2007.02491.x 17635713

[B28] Gunes T. Koklu E. Yikilmaz A. Ozturk M. A. Akcakus M. Kurtoglu S. (2007). Influence of maternal smoking on neonatal aortic intima-media thickness, serum IGF-I and IGFBP-3 levels. Eur. J. Pediatr. 166, 1039–1044. 10.1007/s00431-006-0376-9 17203279

[B29] Hailer Y. D. Montgomery S. M. Ekbom A. Nilsson O. S. Bahmanyar S. (2010). Legg-Calve-Perthes disease and risks for cardiovascular diseases and blood diseases. Pediatrics 125, e1308–e1315. 10.1542/peds.2009-2935 20439602

[B30] Hepner M. Karlaftis V. (2013). Protein C. Methods Mol. Biol. 992, 365–372. 10.1007/978-1-62703-339-8_29 23546729

[B31] Hernández-Zamora E. Rodríguez-Olivas A. O. Rosales-Cruz E. Galicia-Alvarado M. A. Zavala-Hernández C. Reyes-Maldonado E. (2023). Prothrombin time and coagulation factor IX as hemostatic risk markers for legg- calvé-perthes disease. Clin. Appl. Thromb. Hemost. 29, 10760296221151166. 10.1177/10760296221151166 36650707 PMC9869215

[B32] Herring J. A. Kim H. T. Browne R. (2004). Legg-Calve-Perthes disease. Part II: prospective multicenter study of the effect of treatment on outcome. J. Bone Jt. Surg. Am. 86-A, 2121–2134. 15466720

[B33] Huang Q. Li B. Lin C. Chen X. Wang T. Liu J. (2022). MicroRNA sequence analysis of plasma exosomes in early Legg-Calvé-Perthes disease. Cell Signal 91, 110184. 10.1016/j.cellsig.2021.110184 34740784

[B34] Ibrahim T. Little D. G. (2016). The pathogenesis and treatment of legg-calvé-perthes disease. JBJS Rev. 4, e4. 10.2106/jbjs.Rvw.15.00063 27509329

[B35] Jamil K. Zacharin M. Foster B. Donald G. Hassall T. Siafarikas A. (2017). Protocol for a randomised control trial of bisphosphonate (zoledronic acid) treatment in childhood femoral head avascular necrosis due to Perthes disease. BMJ Paediatr. Open 1, e000084. 10.1136/bmjpo-2017-000084 PMC5862235 29637122

[B36] Johnson C. P. Tóth F. Carlson C. S. Armstrong A. R. Zbýň Š. Wu B. (2022). T1ρ and T2 mapping detect acute ischemic injury in a piglet model of Legg-Calvé-Perthes disease. J. Orthop. Res. 40, 484–494. 10.1002/jor.25044 33788301 PMC8481332

[B37] Johnson C. P. Wang L. Tóth F. Aruwajoye O. Carlson C. S. Kim H. K. W. (2018). Quantitative MRI helps to detect hip ischemia: preclinical model of legg-calvé-perthes disease. Radiology 289, 386–395. 10.1148/radiol.2018180497 30063188 PMC6209066

[B38] Jones C. E. Mulpuri K. Teo T. Wilson D. R. d'Entremont A. G. (2022). T1ρ and T2 MRI show hip cartilage damage in adolescents with healed Legg-Calvé-Perthes disease. J. Pediatr. Orthop. B 31, 344–349. 10.1097/bpb.0000000000000892 34139748

[B39] Kallio K. Jokinen E. Raitakari O. T. Hamalainen M. Siltala M. Volanen I. (2007). Tobacco smoke exposure is associated with attenuated endothelial function in 11-year-old healthy children. Circulation 115, 3205–3212. 10.1161/CIRCULATIONAHA.106.674804 17548727

[B40] Kamegaya M. Shinada Y. Akita T. Ogata S. Someya M. Tsuchiya K. (1990). Experimental avascular necrosis of the femoral capital epiphysis and induced subluxation of the hip in young rabbits. J. Pediatr. Orthop. 10, 1–5. 10.1097/01241398-199010010-00001 2298885

[B41] Kamiya N. Kim H. K. (2021). Elevation of proinflammatory cytokine HMGB1 in the synovial fluid of patients with legg-calvé-perthes disease and correlation with *IL-6* . JBMR Plus 5, e10429. 10.1002/jbm4.10429 33615102 PMC7872337

[B42] Kamiya N. Kuroyanagi G. Aruwajoye O. Kim H. K. W. (2019). IL6 receptor blockade preserves articular cartilage and increases bone volume following ischemic osteonecrosis in immature mice. Osteoarthr. Cartil. 27, 326–335. 10.1016/j.joca.2018.10.010 30404032

[B43] Kamiya N. Yamaguchi R. Adapala N. S. Chen E. Neal D. Jack O. (2015a). Legg-Calve-Perthes disease produces chronic hip synovitis and elevation of interleukin-6 in the synovial fluid. J. Bone Min. Res. 30, 1009–1013. 10.1002/jbmr.2435 25556551

[B44] Kamiya N. Yamaguchi R. Aruwajoye O. Adapala N. S. Kim H. K. (2015b). Development of a mouse model of ischemic osteonecrosis. Clin. Orthop. Relat. Res. 473, 1486–1498. 10.1007/s11999-015-4172-6 25666143 PMC4353548

[B45] Kannu P. Irving M. Aftimos S. Savarirayan R. (2011). Two novel *COL2A1* mutations associated with a Legg-Calve-Perthes disease-like presentation. Clin. Orthop. Relat. Res. 469, 1785–1790. 10.1007/s11999-011-1850-x 21442341 PMC3094608

[B46] Kaymaz B. Büyükdogan K. Kaymaz N. Kömürcü E. Golge U. H. Goksel F. (2016). Neutrophil to lymphocyte ratio may be a predictive marker of poor prognosis in Legg-Calvé-Perthes disease. Hip Int. 26, 598–601. 10.5301/hipint.5000381 27229163

[B47] Kenet G. Ezra E. Wientroub S. Steinberg D. M. Rosenberg N. Waldman D. (2008). Perthes' disease and the search for genetic associations: collagen mutations, Gaucher's disease and thrombophilia. J. Bone Jt. Surg. Br. 90, 1507–1511. 10.1302/0301-620x.90b11.20318 18978274

[B48] Kessler J. I. Cannamela P. C. (2018). What are the demographics and epidemiology of legg-calve-perthes disease in a large southern California integrated health system? Clin. Orthop. Relat. Res. 476, 2344–2350. 10.1097/CORR.0000000000000490 30211706 PMC6259889

[B49] Kim H. K. (2011). Legg-Calve-Perthes disease: etiology, pathogenesis, and biology. J. Pediatr. Orthop. 31, S141–S146. 10.1097/BPO.0b013e318223b4bd 21857428

[B50] Kim H. K. Aruwajoye O. Du J. Kamiya N. (2014). Local administration of bone morphogenetic protein-2 and bisphosphonate during non-weight-bearing treatment of ischemic osteonecrosis of the femoral head: an experimental investigation in immature pigs. J. Bone Jt. Surg. Am. 96, 1515–1524. 10.2106/JBJS.M.01361 25232075

[B51] Kim H. K. Bian H. Randall T. Garces A. Gerstenfeld L. C. Einhorn T. A. (2004a). Increased VEGF expression in the epiphyseal cartilage after ischemic necrosis of the capital femoral epiphysis. J. Bone Min. Res. 19, 2041–2048. 10.1359/jbmr.040911 15537448

[B52] Kim H. K. Burgess J. Thoveson A. Gudmundsson P. Dempsey M. Jo C. H. (2016). Assessment of femoral head revascularization in legg-calve-perthes disease using serial perfusion MRI. J. Bone Jt. Surg. Am. 98, 1897–1904. 10.2106/JBJS.15.01477 27852906

[B53] Kim H. K. Morgan-Bagley S. Kostenuik P. (2006). RANKL inhibition: a novel strategy to decrease femoral head deformity after ischemic osteonecrosis. J. Bone Min. Res. 21, 1946–1954. 10.1359/jbmr.060905 17002576

[B54] Kim H. K. Randall T. S. Bian H. Jenkins J. Garces A. Bauss F. (2005). Ibandronate for prevention of femoral head deformity after ischemic necrosis of the capital femoral epiphysis in immature pigs. J. Bone Jt. Surg. Am. 87, 550–557. 10.2106/JBJS.D.02192 15741621

[B55] Kim H. K. Skelton D. N. Quigley E. J. (2004b). Pathogenesis of metaphyseal radiolucent changes following ischemic necrosis of the capital femoral epiphysis in immature pigs. A preliminary report. J. Bone Jt. Surg. Am. 86, 129–135. 10.2106/00004623-200401000-00019 14711955

[B56] Kim H. K. Stephenson N. Garces A. Aya-ay J. Bian H. (2009). Effects of disruption of epiphyseal vasculature on the proximal femoral growth plate. J. Bone Jt. Surg. Am. 91, 1149–1158. 10.2106/JBJS.H.00654 19411464

[B57] Kim H. K. Su P. H. (2002). Development of flattening and apparent fragmentation following ischemic necrosis of the capital femoral epiphysis in a piglet model. J. Bone Jt. Surg. Am. 84-A, 1329–1334. 10.2106/00004623-200208000-00006 12177261

[B58] Kim H. K. Su P. H. Qiu Y. S. (2001). Histopathologic changes in growth-plate cartilage following ischemic necrosis of the capital femoral epiphysis. An experimental investigation in immature pigs. J. Bone Jt. Surg. Am. 83-A, 688–697. 10.2106/00004623-200105000-00007 11379738

[B59] Kim K. M. Wagle S. Moon Y. J. Wang S. I. Park B. H. Jang K. Y. (2018). Interferon β protects against avascular osteonecrosis through interleukin 6 inhibition and silent information regulator transcript-1 upregulation. Oncotarget 9, 3562–3575. 10.18632/oncotarget.23337 29423066 PMC5790483

[B60] Kitoh H. Kitakoji T. Kawasumi M. Ishiguro N. (2008). A histological and ultrastructural study of the iliac crest apophysis in Legg-Calve-Perthes disease. J. Pediatr. Orthop. 28, 435–439. 10.1097/BPO.0b013e318173ed54 18520280

[B61] Koob T. J. Pringle D. Gedbaw E. Meredith J. Berrios R. Kim H. K. (2007). Biomechanical properties of bone and cartilage in growing femoral head following ischemic osteonecrosis. J. Orthop. Res. 25, 750–757. 10.1002/jor.20350 17318897

[B62] Kumasaka Y. Watanabe H. Higashihara T. Kishimoto H. Harada K. Kozuka T. (1991). Changes in the cartilaginous contour of Legg-Calvé-Perthes disease: calculation on T1-weighted MR images. Nihon Igaku Hoshasen Gakkai Zasshi 51, 1232–1239. 1766820

[B63] Kuroyanagi G. Adapala N. S. Yamaguchi R. Kamiya N. Deng Z. Aruwajoye O. (2018). Interleukin-6 deletion stimulates revascularization and new bone formation following ischemic osteonecrosis in a murine model. Bone 116, 221–231. 10.1016/j.bone.2018.08.011 30125727

[B64] Kusumbe A. P. Ramasamy S. K. Adams R. H. (2014). Coupling of angiogenesis and osteogenesis by a specific vessel subtype in bone. Nature 507, 323–328. 10.1038/nature13145 24646994 PMC4943525

[B65] Ladd-Acosta C. Shu C. Lee B. K. Gidaya N. Singer A. Schieve L. A. (2016). Presence of an epigenetic signature of prenatal cigarette smoke exposure in childhood. Environ. Res. 144, 139–148. 10.1016/j.envres.2015.11.014 26610292 PMC4915563

[B66] Lan X. Yu R. Xu J. Jiang X. (2023). Exosomes from chondrocytes overexpressing miR-214-3p facilitate M2 macrophage polarization and angiogenesis to relieve Legg Calvé-Perthes disease. Cytokine 168, 156233. 10.1016/j.cyto.2023.156233 37247447

[B67] Lee J. H. Zhou L. Kwon K. S. Lee D. Park B. H. Kim J. R. (2013). Role of leptin in Legg-Calvé-Perthes disease. J. Orthop. Res. 31, 1605–1610. 10.1002/jor.22415 23832827

[B68] Legg A. T. (2006). An obscure affection of the hip joint. 1910. Clin. Orthop. Relat. Res. 451, 11–13. 10.1097/01.BLO.0000238798.05338.13 17038923

[B69] Li B. Huang Q. Lin C. Lu R. Wang T. Chen X. (2021). Increased circulating CD31+/CD42b-EMPs in Perthes disease and inhibit HUVECs angiogenesis via endothelial dysfunction. Life Sci. 265, 118749. 10.1016/j.lfs.2020.118749 33220290

[B70] Li N. Yu J. Cao X. Wu Q. Y. Li W. W. Li T. F. (2014). A novel p. Gly630Ser mutation of *COL2A1* in a Chinese family with presentations of Legg-Calve-Perthes disease or avascular necrosis of the femoral head. PLoS One 9, e100505. 10.1371/journal.pone.0100505 24949742 PMC4065060

[B71] Little D. G. McDonald M. Sharpe I. T. Peat R. Williams P. McEvoy T. (2005). Zoledronic acid improves femoral head sphericity in a rat model of perthes disease. J. Orthop. Res. 23, 862–868. 10.1016/j.orthres.2004.11.015 16023001

[B72] Liu J. Lin C. Li B. Huang Q. Chen X. Tang S. (2024). Biochanin A inhibits endothelial dysfunction induced by IL-6-stimulated endothelial microparticles in Perthes disease via the NFκB pathway. Exp. Ther. Med. 27, 137. 10.3892/etm.2024.12425 38476892 PMC10928846

[B73] Liu X. Zhang P. Gu Y. Guo Q. Liu Y. (2023). Type H vessels: functions in bone development and diseases. Front. Cell Dev. Biol. 11, 1236545. 10.3389/fcell.2023.1236545 38033859 PMC10687371

[B74] Liu Y. F. Chen W. M. Lin Y. F. Yang R. C. Lin M. W. Li L. H. (2005). Type II collagen gene variants and inherited osteonecrosis of the femoral head. N. Engl. J. Med. 352, 2294–2301. 10.1056/NEJMoa042480 15930420

[B75] Loder R. T. Skopelja E. N. (2011). The epidemiology and demographics of legg-calve-perthes' disease. ISRN Orthop. 2011, 504393. 10.5402/2011/504393 24977062 PMC4063164

[B76] Ma J. Ren Y. Wang B. Sun W. Yue D. Wang W. (2021). Progress of developmental mechanism of subtype H vessels in osteonecrosis of the femoral head. Zhongguo Xiu Fu Chong Jian Wai Ke Za Zhi 35, 1486–1491. 10.7507/1002-1892.202103159 34779178 PMC8586765

[B77] Ma M. Tan Z. Li W. Zhang H. Liu Y. Yue C. (2022). Osteoimmunology and osteonecrosis of the femoral head. Bone Jt. Res. 11, 26–28. 10.1302/2046-3758.111.Bjr-2021-0467.R1 PMC8801166 35045723

[B78] Mastantuono M. Milella P. P. Della Rocca C. Nannerini M. De Paolis M. Larciprete M. (1997). Role of magnetic resonance in the evaluation of the normal and osteochondrosis hip in early and late childhood. Radiol. Med. 94, 571–578. 9524591

[B79] Miashiro E. H. Zanella L. F. Cardoso G. S. Silva G. D. S. de Angelis K. de Almeida S. H. M. (2023). Animal model standardization for studying avascular necrosis of the femoral head in legg-calvé-perthes disease. Rev. Bras. Ortop. (Sao Paulo) 58, e771–e780. 10.1055/s-0042-1749418 37908528 PMC10615593

[B80] Miron R. J. Bohner M. Zhang Y. Bosshardt D. D. (2024). Osteoinduction and osteoimmunology: emerging concepts. Periodontol. 2000 94, 9–26. 10.1111/prd.12519 37658591

[B81] Miyamoto Y. Matsuda T. Kitoh H. Haga N. Ohashi H. Nishimura G. (2007). A recurrent mutation in type II collagen gene causes Legg-Calve-Perthes disease in a Japanese family. Hum. Genet. 121, 625–629. 10.1007/s00439-007-0354-y 17394019

[B82] Mörlin G. B. Hailer Y. D. (2021). High blood pressure and overweight in children with Legg-Calvé-Perthes disease: a nationwide population-based cohort study. BMC Musculoskelet. Disord. 22, 32. 10.1186/s12891-020-03889-9 33407313 PMC7789768

[B83] Mose K. (1980). Methods of measuring in Legg-Calve-Perthes disease with special regard to the prognosis. Clin. Orthop. Relat. Res. 150, 103–109. 10.1097/00003086-198007000-00019 7428206

[B84] Neal D. C. O'Brien J. C. Burgess J. Jo C. Kim H. K. International Perthes Study G. (2015). Quantitative assessment of synovitis in Legg-Calve-Perthes disease using gadolinium-enhanced MRI. J. Pediatr. Orthop. B 24, 89–94. 10.1097/BPB.0000000000000107 25305048

[B85] Perry D. C. Bruce C. E. Pope D. Dangerfield P. Platt M. J. Hall A. J. (2012a). Comorbidities in Perthes' disease: a case control study using the General Practice Research database. J. Bone Jt. Surg. Br. 94, 1684–1689. 10.1302/0301-620X.94B12.29974 23188912

[B86] Perry D. C. Green D. J. Bruce C. E. Pope D. Dangerfield P. Platt M. J. (2012b). Abnormalities of vascular structure and function in children with Perthes disease. Pediatrics 130, e126–e131. 10.1542/peds.2011-3269 22665417

[B87] Perry D. C. Thomson C. Pope D. Bruce C. E. Platt M. J. (2017). A case control study to determine the association between Perthes' disease and the recalled use of tobacco during pregnancy, and biological markers of current tobacco smoke exposure. Bone Jt. J. 99-B, 1102–1108. 10.1302/0301-620X.99B8.BJJ-2016-1282.R1 28768789

[B88] Perthes G. (2012). The classic: on juvenile arthritis deformans. 1910. Clin. Orthop. Relat. Res. 470, 2349–2368. 10.1007/s11999-012-2433-1 22744201 PMC3830075

[B89] Phipps M. C. Huang Y. Yamaguchi R. Kamiya N. Adapala N. S. Tang L. (2016). *In vivo* monitoring of activated macrophages and neutrophils in response to ischemic osteonecrosis in a mouse model. J. Orthop. Res. 34, 307–313. 10.1002/jor.22952 26016440

[B90] Pienkowski D. Resig J. Talwalkar V. Tylkowski C. (2009). Novel three-dimensional MRI technique for study of cartilaginous hip surfaces in Legg-Calvé-Perthes disease. J. Orthop. Res. 27, 981–988. 10.1002/jor.20909 19405084

[B91] Pinheiro M. Dobson C. A. Perry D. Fagan M. J. (2018). New insights into the biomechanics of legg-calve-perthes' disease: the role of epiphyseal skeletal immaturity in vascular obstruction. Bone Jt. Res. 7, 148–156. 10.1302/2046-3758.72.BJR-2017-0191.R1 PMC5895949 29437587

[B92] Pringle D. Koob T. J. Kim H. K. (2004). Indentation properties of growing femoral head following ischemic necrosis. J. Orthop. Res. 22, 122–130. 10.1016/S0736-0266(03)00135-9 14656670

[B93] Qin X. Xi Y. Jiang Q. Chen C. Yang G. (2023). Type H vessels in osteogenesis, homeostasis, and related disorders. Differentiation 134, 20–30. 10.1016/j.diff.2023.09.005 37774549

[B94] Rodríguez-Olivas A. O. Hernández-Zamora E. Reyes-Maldonado E. (2022). Legg-Calvé-Perthes disease overview. Orphanet J. Rare Dis. 17, 125. 10.1186/s13023-022-02275-z 35292045 PMC8922924

[B95] Rush B. H. Bramson R. T. Ogden J. A. (1988). Legg-Calvé-Perthes disease: detection of cartilaginous and synovial change with MR imaging. Radiology 167, 473–476. 10.1148/radiology.167.2.3357958 3357958

[B96] Schlundt C. Saß R. A. Bucher C. H. Bartosch S. Hauser A. E. Volk H. D. (2023). Complex spatio-temporal interplay of distinct immune and bone cell subsets during bone fracture healing. Cells 13, 40. 10.3390/cells13010040 38201244 PMC10777943

[B97] Seguin C. Kassis J. Busque L. Bestawros A. Theodoropoulos J. Alonso M. L. (2008). Non-traumatic necrosis of bone (osteonecrosis) is associated with endothelial cell activation but not thrombophilia. Rheumatol. Oxf. 47, 1151–1155. 10.1093/rheumatology/ken206 18524806

[B98] Spasovski V. Srzentić Dražilov S. Nikčević G. Baščarević Z. Stojiljković M. Pavlović S. (2023). Molecular biomarkers in perthes disease: a review. Diagn. (Basel) 13, 471. 10.3390/diagnostics13030471 PMC9914190 36766577

[B99] Srzentić S. Spasovski V. Spasovski D. Zivković Z. Matanović D. Bascarević Z. (2014). Association of gene variants in TLR4 and *IL-6* genes with Perthes disease. Srp. Arh. Celok. Lek. 142, 450–456. 10.2298/SARH1408450S 25233690

[B100] Stulberg S. D. Cooperman D. R. Wallensten R. (1981). The natural history of Legg-Calve-Perthes disease. J. Bone Jt. Surg. Am. 63, 1095–1108. 10.2106/00004623-198163070-00006 7276045

[B101] Su P. Li R. Liu S. Zhou Y. Wang X. Patil N. (2008). Age at onset-dependent presentations of premature hip osteoarthritis, avascular necrosis of the femoral head, or Legg-Calve-Perthes disease in a single family, consequent upon a p.Gly1170Ser mutation of *COL2A1* . Arthritis Rheum. 58, 1701–1706. 10.1002/art.23491 18512791

[B102] Su P. Zhang L. Peng Y. Liang A. Du K. Huang D. (2010). A histological and ultrastructural study of femoral head cartilage in a new type II collagenopathy. Int. Orthop. 34, 1333–1339. 10.1007/s00264-010-0985-9 20204389 PMC2989094

[B103] Vosmaer A. Pereira R. R. Koenderman J. S. Rosendaal F. R. Cannegieter S. C. (2010). Coagulation abnormalities in Legg-Calve-Perthes disease. J. Bone Jt. Surg. Am. 92, 121–128. 10.2106/JBJS.I.00157 20048104

[B104] Vusal Ismayilov M. (2014). Increased soluble selectins as a ref source J pediatr hematol oncol. Pediatrics. 10.1097/MPH.0000000000000203 25000467

[B105] Wang L. Zhou F. Zhang P. Wang H. Qu Z. Jia P. (2017). Human type H vessels are a sensitive biomarker of bone mass. Cell Death Dis. 8, e2760. 10.1038/cddis.2017.36 28471445 PMC5520742

[B106] Wang S. Zhong H. Ze R. Hong P. Li J. Tang X. (2022). Microarray analysis of lncRNA and mRNA expression profiles in patients with Legg-Calve-Perthes disease. Front. Pediatr. 10, 974547. 10.3389/fped.2022.974547 36160809 PMC9490025

[B107] Wang T. Luo X. Li B. Huang Q. Liu J. Tang S. (2023). Platelet to lymphocyte ratio was a risk factor in Perthes disease. Sci. Rep. 13, 5052. 10.1038/s41598-023-32000-0 36977732 PMC10050405

[B108] Wei W. B. Dang S. J. Li Y. Liu Z. Z. (2017). Alterations of serum osteocalcin levels in patients with Legg-Calvé-Perthes. Hip Int. 27, 92–95. 10.5301/hipint.5000431 27886352

[B109] Woratanarat P. Thaveeratitharm C. Woratanarat T. Angsanuntsukh C. Attia J. Thakkinstian A. (2014). Meta-analysis of hypercoagulability genetic polymorphisms in Perthes disease. J. Orthop. Res. 32, 1–7. 10.1002/jor.22473 23983171

[B110] Xu J. He S. J. Xia T. T. Shan Y. Wang L. (2024). Targeting type H vessels in bone-related diseases. J. Cell Mol. Med. 28, e18123. 10.1111/jcmm.18123 38353470 PMC10865918

[B111] Yamaguchi R. Kamiya N. Adapala N. S. Drissi H. Kim H. K. (2016). HIF-1-Dependent *IL-6* activation in articular chondrocytes initiating synovitis in femoral head ischemic osteonecrosis. J. Bone Jt. Surg. Am. 98, 1122–1131. 10.2106/jbjs.15.01209 27385686

[B112] Yu R. Ma C. Li G. Xu J. Feng D. Lan X. (2023). Inhibition of toll-like receptor 4 signaling pathway accelerates the repair of avascular necrosis of femoral epiphysis through regulating macrophage polarization in perthes disease. Tissue Eng. Regen. Med. 20, 489–501. 10.1007/s13770-023-00529-w 37041432 PMC10219917

[B113] Zhang C. Yang F. Cornelia R. Tang W. Swisher S. Kim H. (2011). Hypoxia-inducible factor-1 is a positive regulator of Sox9 activity in femoral head osteonecrosis. Bone 48, 507–513. 10.1016/j.bone.2010.10.006 20950722

[B114] Zhao Y. Liao S. Lu R. Dang H. Zhao J. Ding X. (2016). Endothelial nitric oxide synthase gene polymorphism is associated with Legg-Calve-Perthes disease. Exp. Ther. Med. 11, 1913–1917. 10.3892/etm.2016.3111 27168827 PMC4840501

[B115] Zheng P. Yang T. Ju L. Jiang B. Lou Y. (2015). Epigenetics in Legg–Calvé–Perthes disease: a study of global DNA methylation. J. Int. Med. Res. 43, 758–764. 10.1177/0300060515591062 26443715

[B116] Zhu H. Qi X. Liu Y. Liao W. Sun X. Tang Y. (2019). The role and underlying mechanisms of microRNA-214 in Legg-Calvé-Perthes disease. Mol. Med. Rep. 20, 685–692. 10.3892/mmr.2019.10271 31180556

